# Supplementary material for: Targeting the Granulocytic Defense against A. fumigatus in Healthy Volunteers and Septic Patients
Source: Int J Mol Sci. 2023 Jun 8;24(12):9911. doi: 10.3390/ijms24129911 (PMC10298267; doi:10.3390/ijms24129911)
Supplement: Supplementary file 1 [file ijms-24-09911-s001.zip › ijms-2404812-supplementary.pdf]

Supplementary Material

(a)

(b)

(c)

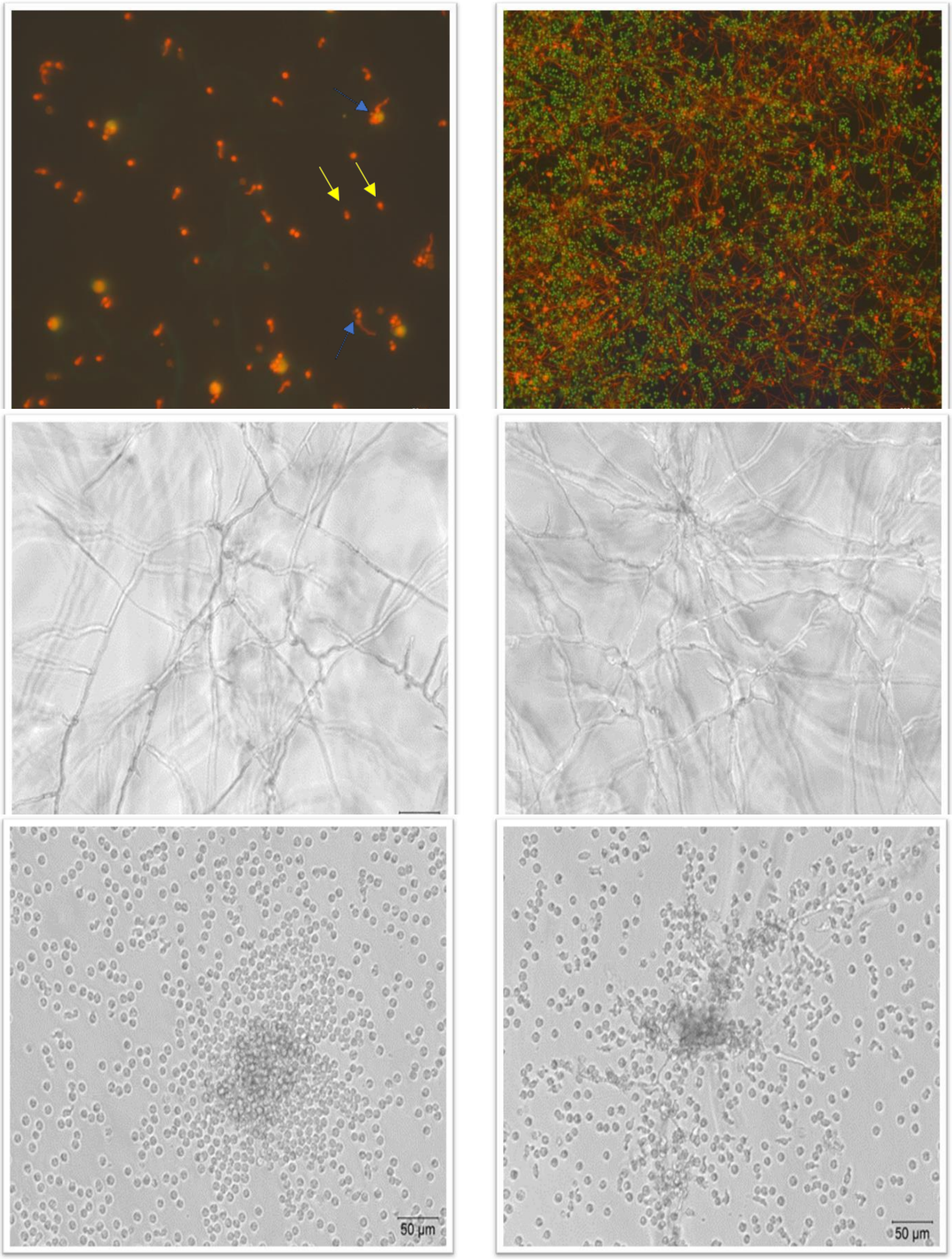

Figure S1. (a) Native microscopy of co-culture with NGs from a healthy participant with calcofluor staining. Left: 40× magnification of 1×10^6 *Aspergillus* conidia incubated with 1×10^5 NGs (E/T ratio 10:1) for 6 h. NGs are stained green-orange and conidia are stained bright orange due to calcofluor staining. Conidia started swelling and changed from round to club-shaped ("germ tubes", yellow arrows). A few conidia started germinating to hyphae (blue arrows). Right: 10× magnification of co-culture of 1×10^6 *Aspergillus* conidia with 2×10^5 NGs (E/T ratio 5:1) for 23 h. Acridine orange stains of RNA (orange) and DNA (green). Green fluorescence was observed in non-vital NGs (DNA) and red fluorescence in vital hyphae and mycelium (RNA). (b) Native microscopy (20× magnification) of *A. fumigatus* only (control) and co-culture of *A. fumigatus* with NGs from one healthy participant after 16 h co-incubation. Left: Control revealed tight mycelium; right: another control with double amount of *A. fumigatus* conidia at the beginning shows even tighter mycelium. (c) Left: E/T ratio of 1280:1 with the same amount of *A. fumigatus* as in (a) resulted in almost no *Aspergillus* growth. Right: E/T ratio of 640:1 with the same amount of *A. fumigatus* conidia at the beginning as in (b). Some mycelium growth can be seen because of insufficient NG inhibition. NG, neutrophil granulocyte; DNA, deoxyribonucleic acid; RNA, ribonucleic acid.
